# Optimized Mahalanobis–Taguchi System for High-Dimensional Small Sample Data Classification

**DOI:** 10.1155/2020/4609423

**Published:** 2020-04-26

**Authors:** Xinping Xiao, Dian Fu, Yu Shi, Jianghui Wen

**Affiliations:** School of Science, Wuhan University of Technology, Wuhan 430070, China

## Abstract

The Mahalanobis–Taguchi system (MTS) is a multivariate data diagnosis and prediction technology, which is widely used to optimize large sample data or unbalanced data, but it is rarely used for high-dimensional small sample data. In this paper, the optimized MTS for the classification of high-dimensional small sample data is discussed from two aspects, namely, the inverse matrix instability of the covariance matrix and the instability of feature selection. Firstly, based on regularization and smoothing techniques, this paper proposes a modified Mahalanobis metric to calculate the Mahalanobis distance, which is aimed at reducing the influence of the inverse matrix instability under small sample conditions. Secondly, the minimum redundancy-maximum relevance (mRMR) algorithm is introduced into the MTS for the instability problem of feature selection. By using the mRMR algorithm and signal-to-noise ratio (SNR), a two-stage feature selection method is proposed: the mRMR algorithm is first used to remove noise and redundant variables; the orthogonal table and SNR are then used to screen the combination of variables that make great contribution to classification. Then, the feasibility and simplicity of the optimized MTS are shown in five datasets from the UCI database. The Mahalanobis distance based on regularization and smoothing techniques (RS-MD) is more robust than the traditional Mahalanobis distance. The two-stage feature selection method improves the effectiveness of feature selection for MTS. Finally, the optimized MTS is applied to email classification of the Spambase dataset. The results show that the optimized MTS outperforms the classical MTS and the other 3 machine learning algorithms.

## 1. Introduction

The Mahalanobis–Taguchi system (MTS) uses the Mahalanobis distance (MD) as a measurement scale and combines Taguchi robust design to achieve system diagnosis and dimension optimization. The MTS is a commonly used multisystem pattern recognition method, which has achieved good results in medical diagnosis [1, 2], financial early warning [3], product detection [4, 5], fault analysis [6], enterprise management, comprehensive evaluation [7], and so on. The MTS is widely applied to the optimization and classification of large sample data or imbalanced data [6, 8–12]. However, in the field of pattern recognition, a large number of recognition problems belong to high-dimensional small sample size problem, and the research on high-dimensional small sample size problem has gradually become a hot spot. For example, image analysis is a typical high-dimensional small sample problem in the field of pattern recognition, and it is also the focus of machine vision field. Image processing is of great significance for machine vision issue and image analysis. The existence of noise in some images makes image processing difficult. In the mentioned images, a speckle noise is the main problem which severely damages the image because of its multiplicative property. Simultaneously, existence of speckle, clutter edge, and image-level clutters can also make false alarms and false detections on retrieval algorithms. It can effectively improve the recognition ability through denoising. Deep learning has a good application in the field of image denoising algorithms. For example, the supervised deep learning method based on deep belief network (DBN) was used to detect changes in synthetic aperture radar (SAR) images [13], a neural network with hybrid algorithm of CNN and multilayer perceptron (CNN-MLP) was suggested for image classification [14], and so on. In addition, image segmentation is the key step from image processing to image analysis. Effective image segmentation method can improve the recognition effect of machine vision [15–17]. Image segmentation is also an important step in texture recognition of images, and the existence of speckle noise will affect image segmentation. Therefore, an algorithm based on wavelet transform and support vector machine was proposed for texture recognition of SAR images [18]. Image processing technology has important applications in image registration [19], coastline detection [20, 21], and so on. Therefore, studying the MTS for high-dimensional small sample data not only provides new ideas for dimension reduction and classification of small sample problems but also extends the application range of the MTS so that the MTS can also play a role in intelligent traffic system [22], image processing, machine vision, and other techniques of electronics field.

The present research on high-dimensional small sample data mainly focuses on three aspects. First, the number of training samples is smaller than that of variables, which will cause the singularity problem of the covariance matrix. Second, when the number of training samples is slightly larger than that of variables, biased eigenvalue estimation will cause the inverse matrix instability of the covariance matrix. Third, the feature selection problem occurs. For the case in which the number of training samples is less than that of features, a common method is to increase the sample size by generating a virtual sample. By using the Monte Carlo method, Karaivanova et al. [23] reconstructed the probability distribution of insufficient data to generate virtual samples. Based on virtual sample generation technology, Gong et al. [24] proposed a new particle swarm optimization (PSO) algorithm to generate effective virtual samples. For the case in which the number of training samples is slightly larger than that of variables, the covariance matrix is optimized mainly from the perspective of eigenvalues. With regard to the poor learning performance of the keep it simple and straightforward (KISS) metric, Tao et al. [25] proposed a double regularization KISS metric learning method for pedestrian recognition problem. Through adjusting the eigenvalues of intraclass and interclass covariance matrices according to the discriminant information of training samples, Liong et al. [26] proposed a new discriminant regularization metric learning method to minimize the estimated distance metric deviation. For the feature selection problem, the classification performance and stability are discussed. Espezua et al. [27] compressed data rapidly and then used an improved projection tracking method to avoid dimension disasters. Hira and Gillies [28] summarized various methods about dimension reduction for high-dimensional microarray data. Kamyab and Eftekhari [29] used a multimodal optimization technology to solve feature selection problems. Goh and Wong [30] proposed a sort-based network algorithm for feature selection in proteomics. To improve the effectiveness and robustness of feature selection technology, Du et al. [31] proposed a hybrid feature selection method based on multicore learning. These methods indicate that the feature selection for high-dimensional data should not only consider the classification performance but also ensure the stability of the results. These studies mainly focused on covariance matrix and feature selection, and few methods can simultaneously solve the problem of dimension reduction and classification for high-dimensional small sample data. Unlike most classification methods, the MTS can screen effective features and construct a classification model by determining threshold. Hence, the MTS can simultaneously solve the problem of dimension reduction and classification.

Many studies have also focused on covariance matrix and feature selection for the MTS. For the covariance matrix, when multiple collinearities occur among variables, the inverse matrix of the covariance matrix does not exist. Taguchi used the Schmidt orthogonalization [32] and the adjoint matrix [33] to calculate MD. Based on Schmidt orthogonalization, Su and Hsiao [34] proposed weighted Schmidt orthogonalization to calculate MD. Shakya et al. [35] used an integrated Schmidt orthogonalization method for the classification of rolling bearings. On the basis of the generalized inverse matrix, Han et al. [36] redefined MD and proposed the Mahalanobis–Taguchi generalized inverse matrix method. Chang et al. [37] used the pseudoinverse of the covariance matrix to calculate MD. Through eliminating multicollinearity by the ridge estimation method, Tao and Cheng [38] proposed the ridge-MD that combines the ridge estimate with MD. For the feature selection, the classical MTS uses the orthogonal table and signal-to-noise ratio (SNR) methods to screen variables. Abraham and Variyath [39] confirmed the possibility of using appropriate algorithms for the dimension reduction and optimization of the MTS. Reséndiz et al. [40] applied the binary ant colony optimization algorithm to optimize the variable combination. Iquebal et al. [41] screened variables on the basis of the maximized degree of dependence between variables and classes or among categories. Reséndiz-Flores et al. [42] used the hybrid binary heuristic algorithm of PSO and gravity search algorithm for feature selection. By introducing chaos mapping and binary particle swarm optimization algorithm, Gu et al. [3] constructed an improved MTS-CBPSO method to screen effective variables. Reyes-Carlos et al. [43] constructed the mathematical model to select features and used metaheuristic algorithms to solve the corresponding model. To solve the feature selection problem of unbalanced welding data, Mahmoud et al. [44] applied the genetic algorithm to the MTS and proposed the Mahalanobis genetic algorithm classifier. Niu and Cheng [45] used optimization model to select variables and constructed probability threshold model for unbalanced data classification. Most of these studies focused on large sample data or unbalanced data, whereas few studies discussed the high-dimensional small sample data. For the covariance matrix, existing research only discussed the multiple collinearity among variables, but few studies discussed the inverse matrix instability of the covariance matrix under the condition of small sample data. For the feature selection, existing research mainly screened features in terms of classification performance, but few studies discussed the instability of feature selection.

In the current work, the optimized MTS for the classification of high-dimensional small sample data is discussed from two aspects, namely, the inverse matrix instability of the covariance matrix and the instability of feature selection. Aimed at the inverse matrix instability problem of the covariance matrix, the Mahalanobis metric based on regularization [25, 46] and smoothing [47, 48] techniques is proposed. Aimed at the instability problem of feature selection, a two-stage feature selection method based on the minimum redundancy-maximum relevance (mRMR) [49, 50] feature selection algorithm and SNR is proposed.

The remainder of this paper is structured as follows. In Section 2, we briefly introduce the implementation steps of the MTS. In Section 3, we construct an optimized MTS model. In Section 4, we select datasets for verification and analysis. In Section 5, we conduct empirical research on the email filtering problem. In Section 6, we derive the conclusion.

## 2. Mahalanobis–Taguchi System

The MTS is a pattern recognition technology based on MD and the Taguchi experiment design. The initial research of the MTS is a two-classification problem. One is set as the normal observations and the other is set as the abnormal observations. To achieve the purpose of system diagnosis and dimension reduction optimization, the orthogonal table and SNR are used to screen the variables, and the classification threshold is determined in accordance with MD.

Assuming the number of the normal observations is *n* and the number of the abnormal observations is *m*, both normal and abnormal observations consist of *p* variables. The *i*^*th*^ observation of the normal observations after normalization is recorded as *Z*_*i*_=[*z*_*i*1_, *z*_*i*2_,…,*z*_*ip*_]_*T*_, *i*=1,2,…, *n*. The abnormal observations are normalized in accordance with the mean and variance of the normal observations, and the *i*^*th*^ observation of the abnormal observations is recorded as *Z*_*i*_=[*z*_*i*1_, *z*_*i*2_,…,*z*_*ip*_]_*T*_, *i*=*n*+1, *n*+2,…, *n*+*m*. The MD from each observation to the reference space can be expressed as

(1) $$\begin{array}{c} {\text{MD}}_{i}=\frac{1}{p}Z_{i}^{T}{\hat{\Sigma }}_{0}^{-1}Z_{i},\;i=1,2,\ldots ,n,n+1,\ldots ,n+m,\\ {\hat{\Sigma }}_{0}=\frac{1}{n-1}\overset{n}{\underset{i=1}{\sum }}Z_{i}Z_{i}^{T},\;i=1,2,\ldots ,n, \end{array}$$

where ${\hat{\Sigma }}_{0}$ is the covariance matrix of the normal observations. In the Mahalanobis–Taguchi system, the standardized variables of the normal observations are used to construct a reference space. The MD of the abnormal observations is significantly larger than that of the normal observations, indicating that the constructed reference space is valid; otherwise, the normal observations should be recollected until a valid reference space is obtained.

The calculation of the traditional MD requires the number of observations is larger than that of variables. Simultaneously, multiple collinearity should be absent among variables to avoid the situation where the inverse matrix of the covariance matrix does not exist. In addition, MD exaggerates the role of variables with minor changes and is susceptible to the instability of the covariance matrix. Therefore, the singularity and instability of the covariance matrix affect the calculation of the traditional MD.

The corresponding two-level orthogonal table *L*_*r*_(2^*p*^), where *r* represents the number of trials, is selected in accordance with the number of initial *p* variables. The level of “1” indicates that the variable is selected, and the level of “2” indicates that the variable is not selected. On the basis of the information of the orthogonal table, the reference space is reconstructed by using the selected variables for each experiment. The MD of each abnormal observation in the new reference space is calculated, and the larger-the-better SNR is calculated as the response value. On the basis of the idea of the experimental design, the effects at different levels of each variable are analyzed, and effective variables are selected. According to the selected variable combination, the MD of each observation is recalculated to determine the threshold by minimizing the classification loss. The unknown observations are then diagnosed.

The classical MTS uses the orthogonal table and SNR to screen variables and select variable combination with high SNR. To get better classification results, the number of training samples should be sufficient to fully reflect the information of each variable. Otherwise, the selected variable combination will not exert a good classification effect on test samples.

## 3. Optimized MTS

This section constructs an optimized MTS for high-dimensional small sample data classification. Firstly, based on the regularization and smoothing techniques, the calculation of the modified Mahalanobis metric is introduced, and the feasibility of the modified Mahalanobis metric is proved. Then, based on the mRMR algorithm and SNR, the implementation steps of the two-stage feature selection method are introduced. Finally, the algorithm flow of the optimized Mahalanobis–Taguchi system is introduced.

### 3.1. Mahalanobis Metric Based on Regularization and Smoothing Techniques

When the number of samples is slightly larger than that of variables, biased eigenvalue estimation will cause the inverse matrix instability of the covariance matrix. Tao et al. [25] proved that the estimation of the covariance matrix is affected by sample size. A small sample size leads to a large generalization bound of covariance matrix estimation. Specifically, the large eigenvalues of the real covariance matrix are overestimated, whereas the small eigenvalues are underestimated. Overestimated large eigenvalues and underestimated small eigenvalues are detrimental to subsequent classification. The calculation of MD depends on the covariance matrix. If the estimation of the covariance matrix is affected, the calculation of MD will produce a deviation. Therefore, the traditional MD is no longer applicable to the high-dimensional small sample data. Because of the one-to-one correspondence between the covariance matrix and a set of eigenvalues or eigenvectors, the estimation performance of the covariance matrix can be improved by improving the estimation of eigenvalues and eigenvectors. On the basis of the above analysis, regularization and smoothing techniques are introduced to improve the performance of covariance estimation in Mahalanobis metric learning.

The correlation coefficient matrix among *p* variables of normal observations is a semipositive matrix, which can be expressed as

(2) $$\begin{array}{c} {\hat{\Sigma }}_{0}=\Phi_{0}\Lambda_{0}\Phi_{0}^{T}, \end{array}$$

where Λ_0_=diag(*λ*_1_, *λ*_2_,…, *λ*_*p*_), with *λ*_*i*_, *i*=1,2,…, *p* being the *i*^*th*^ eigenvalue of ${\hat{\Sigma }}_{0}$, Φ_0_=[*ϕ*_1_, *ϕ*_2_,…, *ϕ*_*p*_], with *ϕ*_*i*_ being the eigenvector corresponding to *λ*_*i*_, and Φ_0_ is an orthogonal matrix.

#### 3.1.1. Smoothing Technique

The basic idea of data smoothing technology is to increase low probability, reduce high probability, and make the probability distribution tend to average. The smoothing technique is introduced to eliminate zero eigenvalues and make the distribution of eigenvalues smooth. However, when all the eigenvalues tend to be the same, the information of the original sample is lost. Therefore, the smoothing technique is used to adjust the small eigenvalues of the covariance matrix.

In accordance with the smoothing technique, a small constant *β*_0_ is used to replace the small eigenvalues of the covariance matrix, which is recorded as

(3) $$\begin{array}{c} \Lambda_{1}=\text{diag}\left(\lambda_{1},\lambda_{2},\ldots ,\lambda_{k},\underset{p-k}{\underset{︸}{\beta_{0}\ldots \beta_{0}}}\right),\;k=0,1,\ldots ,p-1, \end{array}$$

(4) $$\begin{array}{c} \beta_{0}=\frac{1}{p-k}\overset{p}{\underset{j=k+1}{\sum }}\lambda_{j}. \end{array}$$

When the smoothing technology is introduced, some small eigenvalues are replaced with average value. This method not only avoids the appearance of zero eigenvalues but also smooths the distribution of eigenvalues.

#### 3.1.2. Regularization Technique

The basic idea of regularization technology is to use a unit matrix to interpolate the covariance matrix; hence, the sample covariance matrix tends to the unit matrix, which is expressed as

(5) $$\begin{array}{c} {\hat{\Sigma }}_{\gamma }=\left(1-\gamma \right){\hat{\Sigma }}_{0}+\gamma \alpha I=\left(1-\gamma \right)\Phi_{0}\Lambda_{0}\Phi_{0}^{T}+\gamma \alpha \Phi_{0}\Phi_{0}^{T}=\Phi_{0}\left[\left(1-\gamma \right)\Lambda_{0}+\gamma \alpha I\right]\Phi_{0}^{T}, \end{array}$$

where $\alpha =\left(1/p\right)\text{tr}\left({\hat{\Sigma }}_{0}\right)$, 0 < *γ* < 1.

After the introduction of the regularization technique, the eigenvalue corresponding to the covariance matrix becomes

(6) $$\begin{array}{c} \Lambda_{2}=\left(1-\gamma \right)\Lambda_{0}+\gamma \alpha I=\text{diag}\left(\left(1-\gamma \right)\lambda_{1}+\gamma \alpha ,\left(1-\gamma \right)\lambda_{2}+\gamma \alpha ,\ldots ,\left(1-\gamma \right)\lambda_{p}+\gamma \alpha \right). \end{array}$$

The large eigenvalues of the original covariance matrix decrease because of the existence of parameter *γ*. Therefore, parameter *γ* can make ${\hat{\Sigma }}_{0}$ tend to the unit matrix and restrain the overestimation of large eigenvalues.

#### 3.1.3. Mahalanobis Metric Based on Regularization and Smoothing Techniques

For a limited training samples, the estimation of the covariance matrix produces deviations, and the calculation of the traditional MD is affected. In view of the one-to-one correspondence between the covariance matrix and eigenvalues or eigenvectors, the performance of the covariance matrix can be improved by adjusting the eigenvalues, that is, reducing the overestimated large eigenvalues and increasing the underestimated small eigenvalues. Regularization and smoothing technologies are thus introduced into the calculation of MD under the condition of limited samples. Smoothing technology is used to improve the estimation of small eigenvalues, and regularization technology is used to reduce the influence of overestimated large eigenvalues.

The sample covariance matrix is processed by regularization and smoothing techniques, and the new estimation is as follows:

(7) $$\begin{array}{c} {\hat{\Sigma }}_{\gamma ,k}^{\prime }=\Phi_{0}\Lambda_{\gamma ,k}\Phi_{0}^{T}, \end{array}$$

where

(8) $$\begin{array}{c} \Lambda_{\gamma ,k}=\text{diag}\left(\left(1-\gamma \right)\lambda_{1}+\gamma \alpha ,\left(1-\gamma \right)\lambda_{2}+\gamma \alpha ,\ldots ,\left(1-\gamma \right)\lambda_{k}+\gamma \alpha ,\left(1-\gamma \right)\beta_{0}+\gamma \alpha ,\ldots ,\left(1-\gamma \right)\beta_{0}+\gamma \alpha \right). \end{array}$$

The calculation of Mahalanobis distance based on regularization and smoothing techniques (RS-MD) of each sample is transformed into

(9) $$\begin{array}{c} {\text{MD}}_{i}^{\prime }=\frac{1}{p}Z_{i}^{T}{{\hat{\Sigma }}_{\gamma ,k}^{\prime }}^{-1}Z_{i},\;i=1,2,\ldots ,n. \end{array}$$

**Theorem 1.**

The observation is assumed to have an upper bound, that is, ∀*Z*_*i*_ ∈ *Z*, ‖*Z*_*i*_‖ ≤ *Ω*_*Z*_. For any two samples *Z*_*i*_ and *Z*_*j*_ standardized in the same category, we have

(10) $$\begin{array}{c} \left|{\text{MD}}_{i}^{\prime }-{\text{MD}}_{j}^{\prime }\right|\leq \frac{2}{p}\Omega_{Z}^{2}\sqrt{\overset{p}{\underset{i=1}{\sum }}\frac{1}{{\left({\left(\Lambda_{\gamma ,k}\right)}_{ii}\right)}^{2}}}, \end{array}$$

where (Λ_*γ*,*k*_)_*ii*_ represents the *i*^*th*^ diagonal element of Λ_*γ*,*k*_.

**Proof**

From equation (9),

(11) $$\begin{array}{c} \left|{\text{MD}}_{i}^{\prime }-{\text{MD}}_{j}^{\prime }\right|=\left|\frac{1}{p}Z_{i}^{\text{T}}{\hat{\Sigma }}_{\gamma ,k}^{\prime }{\;}^{-1}Z_{i}-\frac{1}{p}Z_{j}^{\text{T}}{\hat{\Sigma }}_{\gamma ,k}^{\prime }{\;}^{-1}Z_{j}\right|=\frac{1}{p}\left|\text{trace}\left(Z_{i}^{\text{T}}{\hat{\Sigma }}_{\gamma ,k}^{\prime }{\;}^{-1}Z_{i}\right)-\text{trace}\left(Z_{j}^{\text{T}}{\hat{\Sigma }}_{\gamma ,k}^{\prime }{\;}^{-1}Z_{j}\right)\right|=\frac{1}{p}\left|\text{trace}\left({\hat{\Sigma }}_{\gamma ,k}^{\prime }{\;}^{-1}Z_{i}Z_{i}^{\text{T}}\right)-\text{trace}\left({\hat{\Sigma }}_{\gamma ,k}^{\prime }{\;}^{-1}Z_{j}Z_{j}^{\text{T}}\right)\right|=\frac{1}{p}\left|\text{trace}\left[{\hat{\Sigma }}_{\gamma ,k}^{\prime }{\;}^{-1}\left(Z_{i}Z_{i}^{\text{T}}-Z_{j}Z_{j}^{\text{T}}\right)\right]\right|\leq \frac{1}{p}\left|\text{trace}\left(\left\|{\hat{\Sigma }}_{\gamma ,k}^{\prime }{\;}^{-1}\right\|\left\|Z_{i}Z_{i}^{\text{T}}-Z_{j}Z_{j}^{\text{T}}\right\|\right)\right|=\frac{1}{p}\left\|{\hat{\Sigma }}_{\gamma ,k}^{\prime }{\;}^{-1}\right\|\left\|Z_{i}Z_{i}^{\text{T}}-Z_{j}Z_{j}^{\text{T}}\right\|. \end{array}$$

Given ‖*Z*_*i*_‖ ≤ *Ω*_*Z*_, we determine

(12) $$\begin{array}{c} \left\|Z_{i}Z_{i}^{\text{T}}\right\|\leq \left\|Z_{i}\right\|\left\|Z_{i}^{\text{T}}\right\|=\Omega_{Z}^{2},\\ \left\|Z_{i}Z_{i}^{\text{T}}-Z_{j}Z_{j}^{\text{T}}\right\|\leq \left\|Z_{i}\right\|\left\|Z_{i}^{\text{T}}\right\|+\left\|Z_{j}\right\|\left\|Z_{j}^{\text{T}}\right\|=2\Omega_{Z}^{2}. \end{array}$$

With

(13) $$\begin{array}{c} \left\|{{\hat{\Sigma }}_{\gamma ,k}^{\prime }}^{-1}\right\|=\sqrt{\text{trace}\left[\left({{\hat{\Sigma }}_{\gamma ,k}^{\prime }}^{-1}\right){\left({{\hat{\Sigma }}_{\gamma ,k}^{\prime }}^{-1}\right)}^{T}\right]}=\sqrt{\text{trace}\left[\left(\Phi_{0}\Lambda_{\gamma ,k}^{-1}\Phi_{0}^{T}\right){\left(\Phi_{0}\Lambda_{\gamma ,k}^{-1}\Phi_{0}^{T}\right)}^{T}\right]}=\sqrt{\text{trace}\left[\Phi_{0}{\left(\Lambda_{\gamma ,k}^{-1}\right)}^{2}\Phi_{0}^{T}\right]}\\ =\sqrt{\text{trace}\left[{\left(\Lambda_{\gamma ,k}^{-1}\right)}^{2}\Phi_{0}^{T}\Phi_{0}\right]}=\sqrt{\text{trace}{\left(\Lambda_{\gamma ,k}^{-1}\right)}^{2}}=\sqrt{\overset{p}{\underset{i=1}{\sum }}\frac{1}{{\left({\left(\Lambda_{\gamma ,k}\right)}_{ii}\right)}^{2}}}, \end{array}$$

we yield

(14) $$\begin{array}{c} \left|{\text{MD}}_{i}^{\prime }-{\text{MD}}_{j}^{\prime }\right|\leq \frac{2}{p}\Omega_{Z}^{2}\sqrt{\overset{p}{\underset{i=1}{\sum }}\frac{1}{{\left({\left(\Lambda_{\gamma ,k}\right)}_{ii}\right)}^{2}}}. \end{array}$$

Theorem 1 indicates that for any two samples from the same class, the upper bound of the difference of RS-MD or MD is related to the eigenvalue of the covariance matrix. Adjusting eigenvalues can improve the performance of the covariance matrix and thus improve the robustness of MD.

**Theorem 2.**

Let (Λ_0_)_*ii*_, (Λ_1_)_*ii*_, and (Λ_2_)_*ii*_ denote the *i*^*th*^ diagonal elements of Λ_0_, Λ_1_, and Λ_2_, respectively,

(15) $$\begin{array}{c} \Psi_{0}=\overset{p}{\underset{i=1}{\sum }}\frac{1}{{\left({\left(\Lambda_{0}\right)}_{ii}\right)}^{2}},\\ \Psi_{1}=\overset{p}{\underset{i=1}{\sum }}\frac{1}{{\left({\left(\Lambda_{1}\right)}_{ii}\right)}^{2}},\\ \Psi_{2}=\overset{p}{\underset{i=1}{\sum }}\frac{1}{{\left({\left(\Lambda_{2}\right)}_{ii}\right)}^{2}},\\ \Psi_{\gamma ,k}=\overset{p}{\underset{i=1}{\sum }}\frac{1}{{\left({\left(\Lambda_{\gamma ,k}\right)}_{ii}\right)}^{2}}, \end{array}$$

and then

(16) $$\begin{array}{c} \Psi_{1}\leq \Psi_{0},\\ \Psi_{\gamma ,k}\leq \Psi_{2}\leq \Psi_{0}. \end{array}$$

**Proof**

The eigenvalue of the sample covariance matrix becomes equation (3) by processing with the smoothing technique. Then,

(17) $$\begin{array}{c} \Psi_{1}=\overset{p}{\underset{i=1}{\sum }}\frac{1}{{\left({\left(\Lambda_{1}\right)}_{ii}\right)}^{2}}=\overset{k}{\underset{i=1}{\sum }}\frac{1}{\lambda_{i}^{2}}+\overset{p}{\underset{i=k+1}{\sum }}\frac{1}{\beta_{0}^{2}}=\overset{k}{\underset{i=1}{\sum }}\frac{1}{\lambda_{i}^{2}}+{\overset{p}{\underset{i=k+1}{\sum }}\left(\frac{1}{\left(1/p-k\right)\sum_{i=k+1}^{p}\lambda_{i}}\right)}^{2}\\ \leq \overset{k}{\underset{i=1}{\sum }}\frac{1}{\lambda_{i}^{2}}+{\overset{p}{\underset{i=k+1}{\sum }}\left(\frac{{\left(p-k\right)}^{2}}{\left(1/p-k\right)\sum_{i=k+1}^{p}\lambda_{i}}\right)}^{2}=\overset{k}{\underset{i=1}{\sum }}\frac{1}{\lambda_{i}^{2}}+{\overset{p}{\underset{i=k+1}{\sum }}\left(\frac{{\left(p-k\right)}^{2}/\sum_{i=k+1}^{p}\lambda_{i}}{\left(p-k\right)}\right)}^{2}\\ \leq \overset{k}{\underset{i=1}{\sum }}\frac{1}{\lambda_{i}^{2}}+\overset{p}{\underset{i=k+1}{\sum }}{\left(\frac{\sum_{i=k+1}^{p}\left(1/\lambda_{i}\right)}{\left(p-k\right)}\right)}^{2}=\overset{k}{\underset{i=1}{\sum }}\frac{1}{\lambda_{i}^{2}}+\left(p-k\right){\left(\frac{\sum_{i=k+1}^{p}\left(1/\lambda_{i}\right)}{\left(p-k\right)}\right)}^{2}\\ \leq \overset{k}{\underset{i=1}{\sum }}\frac{1}{\lambda_{i}^{2}}+\overset{p}{\underset{i=k+1}{\sum }}\frac{1}{\lambda_{i}^{2}}=\overset{p}{\underset{i=1}{\sum }}\frac{1}{\lambda_{i}^{2}}=\overset{p}{\underset{i=1}{\sum }}\frac{1}{{\left({\left(\Lambda_{0}\right)}_{ii}\right)}^{2}}=\Psi_{0}. \end{array}$$

The eigenvalue of the sample covariance matrix becomes equation (6) by processing with the regularization technique. Then,

(18) $$\begin{array}{c} \Psi_{2}=\overset{p}{\underset{i=1}{\sum }}\frac{1}{{\left({\left(\Lambda_{2}\right)}_{ii}\right)}^{2}}=\overset{p}{\underset{i=1}{\sum }}\frac{1}{{\left[\left(1-\gamma \right)\lambda_{i}+\gamma \alpha \right]}^{2}}. \end{array}$$

We assume that

(19) $$\begin{array}{c} g\left(\gamma \right)=\overset{p}{\underset{i=1}{\sum }}\frac{1}{{\left[\left(1-\gamma \right)\lambda_{i}+\gamma \alpha \right]}^{2}},\;0<\gamma <1,\\ \alpha =\left(\frac{1}{p}\right)\text{tr}\left(\hat{\Sigma }\right), \end{array}$$

which yields

(20) $$\begin{array}{c} g^{\prime }\left(\gamma \right)=\overset{p}{\underset{i=1}{\sum }}\frac{-2\left(\alpha -\lambda_{i}\right)}{{\left[\lambda_{i}+\left(\alpha -\lambda_{i}\right)\gamma \right]}^{3}},\\ g^{\prime \prime }\left(\gamma \right)=\overset{p}{\underset{i=1}{\sum }}\frac{6{\left(\alpha -\lambda_{i}\right)}^{2}}{{\left[\lambda_{i}+\left(\alpha -\lambda_{i}\right)\gamma \right]}^{4}}>0. \end{array}$$

Accordingly, *g*′(*γ*) is monotonically increasing in *γ* ∈ (0,1), and

(21) $$\begin{array}{c} g^{\prime }\left(\gamma \right)<g^{\prime }\left(1\right)={\overset{p}{\underset{i=1}{\sum }}\left.\frac{-2\left(\alpha -\lambda_{i}\right)}{{\left[\lambda_{i}+\left(\alpha -\lambda_{i}\right)\gamma \right]}^{3}}\right|}_{\gamma =1}=\overset{p}{\underset{i=1}{\sum }}\frac{-2\left(\alpha -\lambda_{i}\right)}{\alpha^{3}}=0. \end{array}$$

By contrast, *g*(*γ*) is monotonically decreasing in *γ* ∈ (0,1), and

(22) $$\begin{array}{c} \Psi_{2}=g\left(\gamma \right)<g\left(0\right)=\overset{p}{\underset{i=1}{\sum }}\frac{1}{\lambda_{i}^{2}}=\overset{p}{\underset{i=1}{\sum }}\frac{1}{{\left({\left(\Lambda_{0}\right)}_{ii}\right)}^{2}}=\Psi_{0}. \end{array}$$

The eigenvalue of the sample covariance matrix becomes equation (8) by processing with the regularization and smoothing techniques. Then,

(23) $$\begin{array}{c} \Psi_{\gamma ,k}=\overset{p}{\underset{i=1}{\sum }}\frac{1}{{\left({\left(\Lambda_{\gamma ,k}\right)}_{ii}\right)}^{2}}=\overset{k}{\underset{i=1}{\sum }}\frac{1}{{\left[\left(1-\gamma \right)\lambda_{i}+\gamma \alpha \right]}^{2}}+\overset{p}{\underset{i=k+1}{\sum }}\frac{1}{{\left[\left(1-\gamma \right)\beta_{0}+\gamma \alpha \right]}^{2}}\\ \leq \overset{k}{\underset{i=1}{\sum }}\frac{1}{{\left[\left(1-\gamma \right)\lambda_{i}+\gamma \alpha \right]}^{2}}+\overset{p}{\underset{i=k+1}{\sum }}\frac{1}{{\left[\left(1-\gamma \right)\lambda_{i}+\gamma \alpha \right]}^{2}}=\overset{p}{\underset{i=1}{\sum }}\frac{1}{{\left[\left(1-\gamma \right)\lambda_{i}+\gamma \alpha \right]}^{2}}=\Psi_{2}. \end{array}$$

Thus,

(24) $$\begin{array}{c} \Psi_{1}\leq \Psi_{0},\\ \Psi_{\gamma ,k}\leq \Psi_{2}\leq \Psi_{0}. \end{array}$$

Theorem 2 reflects the relationship between the calculated value of each eigenvalue sequence when the eigenvalues are processed by different methods. Combining Theorems 1 and 2, we know that for any two samples in the same class, the upper bound of the difference fluctuation of RS-MD is smaller than that of traditional MD. Hence, the Mahalanobis metric based on regularization and smoothing techniques is more robust than the traditional Mahalanobis metric.

### 3.2. Two-Stage Feature Selection Algorithm Based on mRMR Algorithm and SNR

High-dimensional small sample data can cause the instability problem of feature selection. When the training samples produce a small disturbance, the selected variable combination may produce a large difference. High-dimensional small sample data often contain a large number of redundant, uncorrelated, and noise features. They cannot fully reflect the feature information due to the small number of training samples, thereby resulting in great differences in the selection of feature combination for different training samples. The MTS screens variables only from the perspective of classification accuracy. However, for limited training samples, the selected variable combination based on classification accuracy is no longer reliable. This paper accordingly proposes a two-stage feature selection method based on the mRMR algorithm and SNR. First, the mRMR algorithm is used to remove redundant and noise features, and the feature which is highly relevant to class labels is selected. Then, in accordance with the orthogonal table and SNR, a feature subset with strong resolution is selected to achieve the goals of robust optimization and dimension reduction.

#### 3.2.1. One-Time Feature Selection Based on mRMR Algorithm

High-dimensional small sample data contain a large number of redundant, uncorrelated, and noisy features, which not only increase computational complexity significantly and reduce the performance of the classifier but also cause the instability of feature selection. Therefore, the mRMR algorithm is introduced to ensure the validity of the selected features.

According to the cost functions of information difference and information entropy, the mRMR algorithm is aimed at measuring the maximal sample information and minimal relevance among features. The correlation between features and categories or features is measured by mutual information [51]. Mutual information is a measure of the degree of interdependence between two random variables. Extensive mutual information between two random variables indicates a strong correlation between them [52].

The number of samples *n*, the number of features *p*, and category *c* of dataset *X* are given. The features are recorded as *a*_1_, *a*_2_,…, *a*_*p*_. The value range of feature *a*_*i*_ is *V*_*i*_, and the value range of category *c* is *V*_*c*_.

The mutual information *I*(*a*_*i*_, *c*) between feature *a*_*i*_ and category *c* is

(25) $$\begin{array}{c} I\left(a_{i},c\right)=\underset{v_{i}\in V_{i}}{\sum }\underset{v_{c}\in V_{c}}{\sum }p\left(v_{i},v_{c}\right)\log\frac{p\left(v_{i},v_{c}\right)}{p\left(v_{i}\right)p\left(v_{c}\right)}, \end{array}$$

where *p*(*v*_*i*_, *v*_*c*_) represents the probability that the value of feature *a*_*i*_ is *v*_*i*_ and the value of class *c* is *v*_*c*_. A large value of *I*(*a*_*i*_, *c*) shows a high degree of association between feature *a*_*i*_ and category *c* [53].

The mutual information *I*(*a*_*i*_, *a*_*j*_) between features *a*_*i*_ and *a*_*j*_ is

(26) $$\begin{array}{c} I\left(a_{i},a_{j}\right)=\underset{v_{i}\in V_{i}}{\sum }\underset{v_{j}\in V_{j}}{\sum }p\left(v_{i},v_{j}\right)\log\frac{p\left(v_{i},v_{j}\right)}{p\left(v_{i}\right)p\left(v_{j}\right)}, \end{array}$$

where *p*(*v*_*i*_, *v*_*j*_) represents the probability that the value of feature *a*_*i*_ is *v*_*i*_ and the value of feature *a*_*j*_ is *v*_*j*_. A large value of *I*(*a*_*i*_, *a*_*j*_) implies a high similarity of feature *a*_*i*_ to feature *a*_*j*_ [53].

The maximum correlation and minimum redundancy of the mRMR algorithm are calculated as follows:

(27) $$\begin{array}{c} \max\;D\left(V,c\right):D=\frac{1}{\left|V\right|}\underset{a_{i}\in V}{\sum }I\left(a_{i},c\right),\\ \min R\left(V\right):R=\frac{1}{{\left|V\right|}^{2}}\underset{a_{i},a_{0}\in V}{\sum }I\left(a_{i},a_{0}\right). \end{array}$$

where *V* and |*V*| represent the feature subset and its dimension, respectively; *D* represents the mean of the mutual information; and *R* represents the mutual information among features [49].

The mRMR algorithm generates features with minimum redundancy and maximum correlation through the following two criteria:

(28) $$\begin{array}{c} \max\Phi_{1}\left(D,R\right):\Phi_{1}=D-R,\\ \max\Phi_{2}\left(D,R\right):\Phi_{2}=D/R. \end{array}$$

#### 3.2.2. Secondary Feature Selection Based on SNR

The mRMR algorithm removes the redundant and noisy features, and the robustness of feature selection is guaranteed. However, the use of this algorithm does not mean that the features in the obtained feature subset are beneficial to the classification. The features that make great contribution to the classification accuracy is further filtered by using orthogonal table and SNR.

A suitable two-level orthogonal table is selected on the basis of the selected feature subset by the mRMR algorithm. According to the information of the orthogonal table, the reference space is reconstructed by using the selected features for each experiment, and the RS-MD of each abnormal observation is calculated in accordance with equation (9). At this point, the calculation of the larger-the-better SNR is as follows:

(29) $$\begin{array}{c} \text{SN}=-10\mathrm{lg}\;\left(\frac{1}{m}\overset{n+m}{\underset{i=n+1}{\sum }}\frac{1}{{\text{MD}}_{i}^{\prime }}\right). \end{array}$$

For variable *x*_*j*_, $\bar{{\text{SN}}_{j}^{+}}$ is used to represent the SNR mean when this variable is used; $\bar{{\text{SN}}_{j}^{-}}$ is used to represent the SNR mean when this variable is not used; and $\Delta =\bar{{\text{SN}}_{j}^{+}}-\bar{{\text{SN}}_{j}^{-}}$ represents the SNR increment. When the increment is positive, variable *x*_*j*_ is retained; otherwise, variable *x*_*j*_ is removed. The contribution degree of each variable to the classification accuracy is judged on the basis of the increment in SNR, and the feature combination with a large contribution degree is selected.

The two-stage feature selection not only ensures the robustness of the selected feature combination by using the mRMR algorithm but also improves the classification accuracy by using the orthogonal table and SNR. Therefore, it achieves the goals of robust optimization and dimension reduction The optimized Mahalanobis–Taguchi system uses the Mahalanobis distance based on regularization and smoothing techniques (RS-MD) as a measurement scale and uses the two-stage feature selection method to screen features. The algorithm flow of the optimized Mahalanobis–Taguchi system is presented in Algorithm 1.

## 4. Effectiveness Verification of Optimized MTS

To verify the robustness of the RS-MD and the validity of the two-stage feature selection method, five datasets from the UCI database are shown in this section. The MTS uses normal observations to construct the reference space, and the information of the reference space is used to calculate the covariance matrix and MD. To satisfy the characteristics of high-dimensional small sample data, the number of samples cannot exceed 10 times the number of features. Data processing is conducted on the selected five datasets to remove missing values and undifferentiated variables. The information obtained is shown in Table 1.

### 4.1. Comparative Analysis of Traditional MD and RS-MD

**Figure 1:**
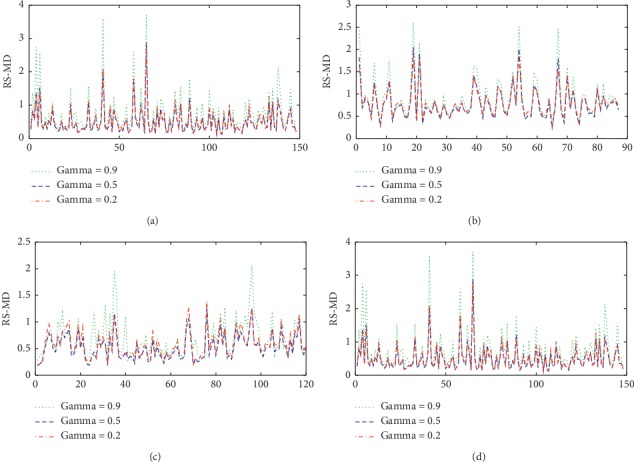
The distribution of RS-MD for normal observations. (a) Ionosphere. (b) Z-Alizadeh Sani. (c) Parkinson dataset with replicated acoustic features. (d) Breast Cancer Wisconsin (prognostic).

The calculation of traditional Mahalanobis distance requires that the covariance matrix is not singular, that is, the number of normal observations is larger than that of features. According to the information of normal observations in Ionosphere, Z-Alizadeh Sani, Parkinson dataset with replicated acoustic features, and Breast Cancer Wisconsin (prognostic) datasets, the benchmark space is constructed and the data are standardized. The MD of each sample in the above datasets is calculated. Because the RS-MD is affected by parameters *β*_0_ and *γ*, we choose to smooth the eigenvalues less than 0.01. Different parameters *γ* are also selected for discussion. When parameter *γ* is taken as 0.2, 0.5, and 0.9, the RS-MD under each dataset is calculated. The calculation results are shown in Figure 1.

Figure 1 shows the distribution of RS-MD for normal observations of each dataset when parameter *γ* is taken as 0.2, 0.5, and 0.9. When the parameter *γ* is 0.2 or 0.5, the fluctuations of the calculated RS-MD are minimal, thereby indicating that the results are highly robust when the parameter *γ* is small. When the parameter *γ* is 0.9, the fluctuations of the calculated RS-MD become large, indicating that the robustness of the results is weakened when the parameter *γ* is large. To further reflect the influence of the parameter *γ* on the calculation results, the variance of RS-MD when the parameter *γ* takes different values is shown in Table 2.

**Figure 2:**
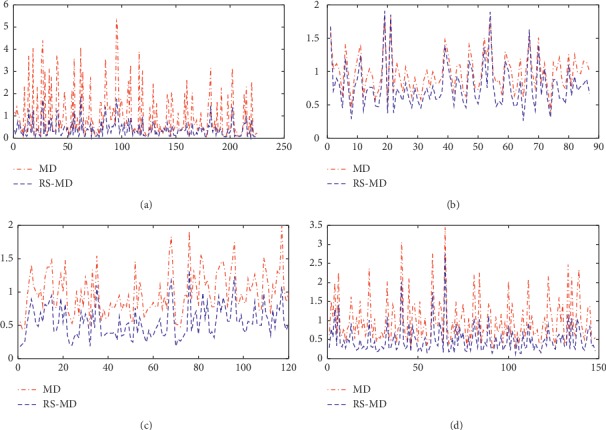
Comparison of the distributions between MD and RS-MD. (a) Ionosphere. (b) Z-Alizadeh Sani. (c) Parkinson dataset with replicated acoustic features. (d) Breast Cancer Wisconsin (prognostic).

Table 2 shows the variance of the RS-MD in the normal observations when parameter *γ* is taken as 0.2, 0.3, 0.5, and 0.9. When parameter *γ* is less than 0.5, the variance of the calculated RS-MD is small, indicating that the fluctuation is small. When parameter *γ* is 0.9, the variance of the calculated RS-MD increases gradually. Hence, when parameter *γ* approaches 1, the fluctuation of the RS-MD increases and the robustness decreases. This is because the eigenvalues of the estimated covariance matrix at this time are almost equal, thereby resulting in an overfitting problem. The comparison of the variances shows that the comprehensive effect is better when parameter *γ* is 0.3. Therefore, we set parameter *γ* as 0.3 and smooth the eigenvalues less than 0.01. The calculation results of RS-MD and traditional MD are shown in Figure 2.

Figure 2 depicts the distributions of MD and RS-MD for the normal observations of each dataset. The distributions of between MD and RS-MD in the Z-Alizadeh Sani dataset are relatively close. The RS-MD is slightly smaller than the traditional MD, and the volatility is slightly reduced. In the other three datasets, the RS-MD is smaller than the traditional MD, and the volatility is significantly reduced. Therefore, the Mahalanobis metric based on the regularization and smoothing techniques is more robust than the traditional Mahalanobis metric.

**Figure 3:**
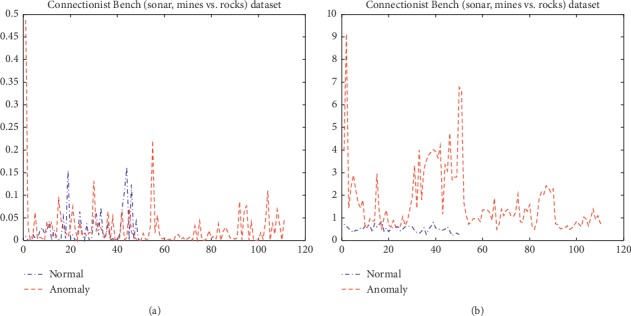
Comparison between RS-MD and GS-MD. (a) GS-MD. (b) RS-MD.

However, when the number of normal observations is smaller than that of features, the calculated sample covariance matrix is singular, and the traditional Mahalanobis distance cannot be calculated. In order to verify the validity of the RS-MD under this condition, the Gram–Schmidt Mahalanobis distance (GS-MD) is compared with the RS-MD. Taking the Connectionist Bench (sonar, mines vs. rocks) dataset as an example, the calculation results are shown in Figure 3. It can be seen from Figure 3 that although GS-MD can calculate the Mahalanobis distance of each sample, the Mahalanobis distance of the samples in two classes almost overlaps, making it difficult to distinguish the samples in two classes effectively. When the RS-MD is used, there is a significant difference in two classes. The RS-MD can be used as an index to distinguish the samples. Therefore, the RS-MD can be used as a metric when the number of normal samples is less than that of features, and the discrimination of the different samples can be improved.

### 4.2. Comparative Analysis between the Two-Stage Feature Selection Method and the Feature Selection of Traditional MTS

To verify the validity of the two-stage feature selection method, the stability and classification accuracy of feature selection are analyzed in this section. The data of each dataset are divided into five folds, four of which are used as training data. In order to measure the stability of feature selection, the Jaccard coefficient is used to calculate the similarity of feature subsets.

The Jaccard coefficient is a common measure of similarity, which is used to measure the similarities among sample sets [54]. For any two sets *A* and *B*, the Jaccard coefficient is defined as follows:

(30) $$\begin{array}{c} J\left(A,B\right)=\frac{\left|A\cap B\right|}{\left|A\cup B\right|}=\frac{\left|A\cap B\right|}{\left|A\right|+\left|B\right|-\left|A\cap B\right|}. \end{array}$$

**Figure 4:**
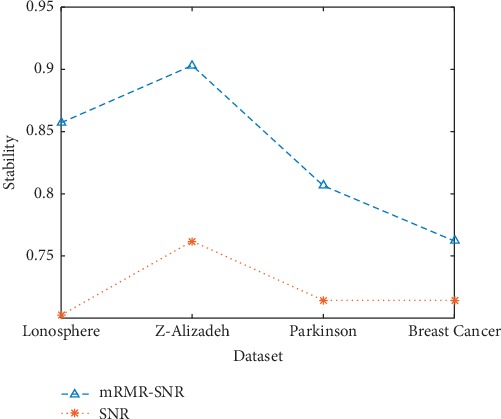
Stability of feature selection results for each dataset.

The mean of Jaccard coefficient in five experiments is taken as a measure of the stability of feature selection. The results are shown in Figure 4. Figure 4 presents the stability of the feature subset obtained by using two feature selection methods in each dataset, where mRMR-SNR represents the two-stage feature selection method and SNR represents the feature selection method of traditional MTS. Compared with the traditional MTS using the SNR to screen variables, the effect of the mRMR-SNR is better. This result shows that the two-stage feature selection method is beneficial to the improvement of the robustness of feature selection.

**Figure 5:**
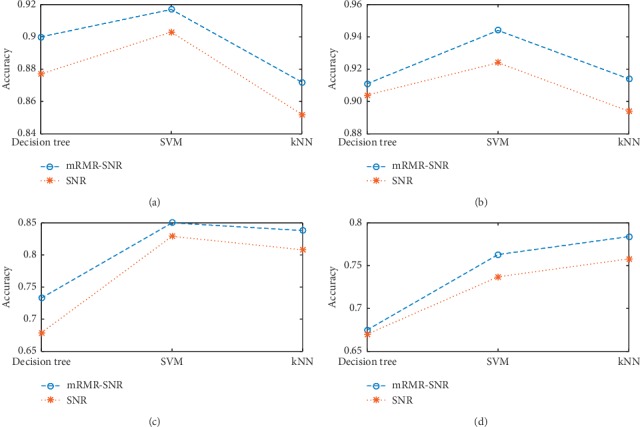
Classification accuracy after feature selection of each dataset. (a) Ionosphere. (b) Z-Alizadeh Sani. (c) Parkinson dataset with replicated acoustic features. (d) Breast Cancer Wisconsin (prognostic).

On the basis of the results of the two feature selection methods, the classification accuracy in each dataset after feature selection is calculated. Decision tree, SVM, and kNN are used as classifiers to measure the classification accuracy. Five-fold cross validation is used to calculate the classification accuracy for each dataset, and the results are shown in Figure 5.

Figure 5 presents the classification accuracy calculated by different classifiers after using two feature selection methods for each dataset. It can be seen that according to the feature subset obtained from mRMR-SNR in each dataset, the classification accuracy calculated is higher. Thus, the two-stage feature selection method is helpful to select the effective features in classifying.

## 5. Empirical Analysis

The formation and development of email offer great convenience to daily life. However, large numbers of spam cases also cause many problems for users and service providers. Therefore, how to obtain effective emails becomes a concern, and email filtering has gradually become an important way [55]. The purpose of email filtering is to distinguish regular messages from spam; this objective belongs to a typical two-class problem. Traditional classification algorithms often require a large number of labeled emails as training samples, but the collection and tagging of a large number of emails greatly increase the cost of consumption. Hence, improving email filtering performance under small sample conditions is an important research issue [56]. The MTS does not depend on the distribution type of data and can achieve classification prediction after reducing dimension. It is a practical pattern recognition and classification prediction method for multidimensional variables. Thus, we apply the optimized MTS to email filtering under small sample conditions.

### 5.1. Data Preprocessing

This section takes the Spambase dataset as an example provided in the UCI database. The dataset contains 4,601 email samples (2,788 regular emails and 1813 spam emails). The text content of each email is described by 56 different variables and 1 attribute variable. A total of 360 emails (190 regular emails and 170 spam emails) are randomly selected from the dataset to make up the training set, and the test set consists of 300 emails (160 regular emails and 140 spam emails). This is aimed at satisfying the requirements of high-dimensional small sample data and improving the efficiency of the algorithm.

### 5.2. Construction of Measurement Scale Based on Modified Mahalanobis Metric

In the training set, 190 regular emails are normal observations and 170 spam emails are abnormal observations. The RS-MD of each observation is calculated by equation (9). According to the results of the verification analysis, parameter *γ* is set as 0.3, and eigenvalues less than 0.01 are smoothed. According to the calculation results, the RS-MD of most of the abnormal observations is bigger than that of the normal observations, whereas the RS-MD of the normal observations are basically concentrated at approximately 1. Thus, the constructed reference space is effective.

### 5.3. Two-Stage Feature Selection Based on mRMR Algorithm and SNR

The original data consist of 56 variables, which are recorded as *X*_1_, *X*_2_,…, *X*_56_. The mRMR algorithm is first used to remove the noise variables and the redundant variables. The correlation is measured by mutual information, which is calculated by equations (25) and (26). The 31 features are retained, and their scores are shown in Table 3.

An orthogonal table *L*_32_(2^31^) is selected on the basis of the 31 selected features. On the basis of the information of the orthogonal table, the RS-MD of each abnormal observation under different feature combinations is recalculated, and the SNR is calculated according to equation (30). The SNR values obtained from each test are shown in Table 4.

Combined with the orthogonal table and SNR, the mean of SNR of each feature at different levels is analyzed. The mean of SNR reflects the effect of the variable at different levels. When the mean of SNR at level 1 is greater than level 2, indicating that using this variable is more advantageous than not using it. That is, those variables are effective variables and are beneficial to classification. Conversely, when the mean of SNR at level 1 is lower than level 2, indicating that the effect of using this variable is lower than not using it. That is, those variables exert a slight influence on the classification and can be deleted. For valid variables, the difference of the mean of SNR at different levels reflects the significance of the variable. The greater the difference, the greater the contribution of the variable to the classification. Therefore, the reduced benchmark space is composed of these 15 variables *X*_6_, *X*_10_, *X*_15_, *X*_16_, *X*_18_, *X*_19_, *X*_22_, *X*_23_, *X*_24_, *X*_36_, *X*_38_, *X*_51_, *X*_52_, *X*_53_, *X*_55_.

### 5.4. Threshold Calculation and Classification Prediction

**Figure 6:**
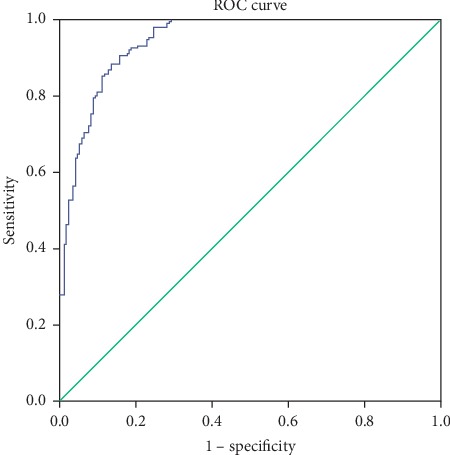
ROC curve corresponding to the Mahalanobis distance of the training set after feature selection.

The reference space is reconstructed in accordance with the 15 variables selected in the feature selection process, and the MD of each sample in the new reference space is calculated. The traditional MD is then used because the data after feature selection are no longer high-dimensional small sample data. Based on the calculated MD, the ROC curve is used to determine the threshold of the system. The result is shown in Figure 6. When the threshold is 1.9377, the classification accuracy of the training set reaches a maximum of 0.9194. The determined threshold is used to classify the test set, and the classification accuracy of the test set is 0.9067 eventually.

### 5.5. Comparison with Common Classification Methods for High-Dimensional Small Sample Data

For the classification problem of high-dimensional small sample data, a feature selection method, such as filter and embedded methods, is first used to filter the variables, and then a common machine learning algorithm is used to classify the dimension-reduced dataset. The relief method and the SVM-RFE method are the commonly used methods in the filter and embedded method, respectively. Therefore, this section first uses the relief and SVM-RFE methods to reduce the dimension of high-dimensional small sample data. Then, the decision tree, SVM, and kNN algorithm are used to classify the reduced-dimensional dataset.

Fifteen variables are selected by the relief or SVM-RFE method, and then the dimension-reduced dataset is classified by decision tree, SVM, and kNN algorithm. The results are compared with those of the optimized MTS. The comparison results are shown in Table 5.

As shown in Table 5, the optimized MTS has better classification effect than the classical MTS for training and test samples. This result shows that compared with the classical MTS, the classification and prediction capability of the optimized MTS is better. That is, the optimized MTS is more suitable for small sample data.

After screening feature by relief and SVM-RFE methods, the classification effect of the SVM algorithm is better than that of the decision tree and kNN algorithm. This result shows that the SVM algorithm has the better classification performance under the condition of small samples. However, the classification effect of the three classifiers is lower than that of the optimized MTS. The optimized MTS has good dimension reduction and classification performance for high-dimensional small sample data. Moreover, the dimension reduction and classification prediction are separated in the commonly used classification methods for high-dimensional small sample data. By contrast, the optimized MTS can complete classification prediction after reducing variables, that is, solve the problem of dimension reduction and classification prediction at the same time. The optimized MTS thus maintains work efficiency to a certain extent.

## 6. Conclusion

This paper proposes the optimized MTS for high-dimensional small sample data. Aimed at the inverse matrix instability problem of the covariance matrix, a Mahalanobis metric based on regularization and smoothing techniques is proposed. Aimed at the feature selection problem, a two-stage feature selection algorithm based on the mRMR algorithm and SNR is proposed. Through the verification analysis of five datasets, the robustness of the modified Mahalanobis metric and the effectiveness of the two-stage feature selection method are verified. The optimized MTS is applied to email filtering problems under small sample conditions and achieves a good classification and dimension reduction effect. Simultaneously, compared with the classical MTS and the commonly used classification algorithms for high-dimensional small sample data, the optimized MTS performs better. Thus, the optimized MTS not only improves the generalization capability of the MTS but also provides a new approach for high-dimensional small sample data.

## Figures and Tables

**Algorithm 1:**
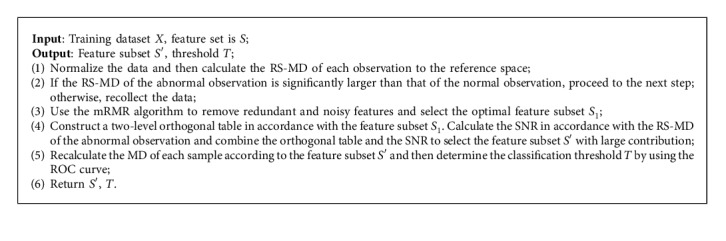
The algorithm flow of optimized Mahalanobis–Taguchi system.

**Table 1:** Description of the dataset.

Dataset name	Number of variables	Number of samples	Positive class	Negative class
Ionosphere	33	351	Good/225	Bad/126
Z-Alizadeh Sani	48	303	Normal/87	CAD/216
Parkinson dataset with replicated acoustic features	46	240	Healthy/120	PD/120
Breast Cancer Wisconsin (prognostic)	34	194	Recurrent/148	Nonrecurrent/46
Connectionist Bench (sonar, mines vs. rocks)	60	161	R/50	M/111

**Table 2:** The variance of the RS-MD.

Dataset name	*γ*=0.2	*γ*=0.3	*γ*=0.5	*γ*=0.9
Ionosphere	0.1555	0.1182	0.0978	0.2463
Z-Alizadeh Sani	0.1127	0.1228	0.1483	0.2633
Parkinson dataset with replicated acoustic features	0.0650	0.0589	0.0552	0.1207
Breast Cancer Wisconsin (prognostic)	0.1419	0.1359	0.1455	0.3499

**Table 3:** Selected feature and its score by mRMR algorithm.

Feature	*X*_18_	*X*_46_	*X*_28_	*X*_52_	*X*_40_	*X*_2_	*X*_26_	*X*_21_	*X*_6_	*X*_43_	*X*_37_
Score	0.000	−0.094	−0.019	−0.007	−0.014	−0.027	−0.026	−0.019	−0.016	−0.017	−0.030
Feature	*X*_41_	*X*_23_	*X*_24_	*X*_45_	*X*_55_	*X*_51_	*X*_32_	*X*_47_	*X*_15_	*X*_38_	*X*_13_
Score	−0.022	−0.028	−0.027	−0.033	−0.041	−0.050	−0.052	−0.060	−0.057	−0.061	−0.066
Feature	*X*_31_	*X*_19_	*X*_22_	*X*_25_	*X*_10_	*X*_53_	*X*_36_	*X*_16_	*X*_48_		
Score	−0.068	−0.063	−0.076	−0.072	−0.085	−0.092	−0.094	−0.096	−0.098		

**Table 4:** Signal-to-noise ratio values under each test.

Test	1	2	3	4	5	6	7	8	9	10	11
SNR	1.513	−1.271	−4.010	0.692	−7.809	−2.428	−3.104	−9.281	−5.210	−6.468	−5.422
Test	12	13	14	15	16	17	18	19	20	21	22
SNR	−5.690	−5.506	−4.236	−3.931	−2.749	−4.634	−4.227	−4.107	−6.069	−0.658	−2.976
Test	23	24	25	26	27	28	29	30	31	32	
SNR	−2.747	0.260	−3.678	−4.094	−4.759	−3.800	−4.712	−3.734	−5.076	−4.008	

**Table 5:** Comparison of results between optimized MTS and the classification methods for high-dimensional small sample data.

	Optimized MTS		Classical MTS		Decision tree	
					Relief	SVM-RFE
Number of features	15		20		15	15
Training set	0.9194		0.8722		0.8333	0.8444
Test set	0.9067		0.8633		0.8433	0.8533

	SVM		kNN			
	Relief	SVM-RFE	Relief	SVM-RFE		

Number of features	15	15	15	15		
Training set	0.8722	0.8944	0.8583	0.8750		
Test set	0.8733	0.8967	0.8600	0.8867		

## Data Availability

The calculation software used in this paper includes MATLAB 2016a, SPSS 22, and Minitab 17. The UCI database is available online at https://archive.ics.uci.edu/ml/datasets.php.
